# Integrin β3 overexpression contributes to podocyte injury through inhibiting RhoA/YAP signaling pathway

**DOI:** 10.1080/21655979.2021.1906097

**Published:** 2021-04-05

**Authors:** Zhuo Li, Zhiwen Lian, Jianchao Ma, Li Zhang, Xingji Lian, Shuangxin Liu, Jianteng Xie, Zhonglin Feng, Ting Lin, Hong Zhang, Xinling Liang

**Affiliations:** Department of Nephrology, Guangdong Provincial People’s Hospital, Guangdong Academy of Medical Sciences, Guangzhou, China

**Keywords:** ITGβ3, high glucose, podocyte, RhoA/YAP pathway

## Abstract

Axis formed by integrin β3 (ITGβ3)-Ras homolog gene family, member A (RhoA), and Yes-associated protein (YAP) plays an important role in atherosclerosis. In addition, ITGβ3 overexpression was noted in high-glucose (HG) exposure podocytes. However, the ITGβ3–RhoA–YAP axis on HG-induced podocyte injury remains unclear. This study aimed to investigate whether ITGβ3 regulates podocyte injury by regulating the RhoA–YAP axis. The function and potential mechanism of ITGβ3 were observed through in vitro wound-healing assays, flow cytometry, reverse transcription-quantitative polymerase chain reaction (RT-qPCR), and western blot assay. Results showed that HG treatment increased the ability of wound closure and apoptosis; however, in spite of HG treatment, ITGβ3 inhibition mitigated the ability of wound closure and apoptosis in podocytes. By contrast, overexpression of ITGβ3 increased the wound closure and apoptosis abilities of podocytes. Under HG treatment, ITGβ3 knockdown is associated with upregulation of RhoA, total YAP1, and nucleus YAP1, whereas ITGβ3 overexpression has opposite effect. In addition, RhoA overexpression in podocytes reverses the effect of ITGβ3 overexpression on the wound closure and apoptosis abilities of podocytes, rescue the expression of YAP in ITGβ3 overexpression podocytes. Taken together, ITGβ3 overexpression promotes podocytes injury by inhibiting RhoA-YAP axis. This will provide a new clue for preventing podocyte from damage.

## Graphical abstract

**Figure:**
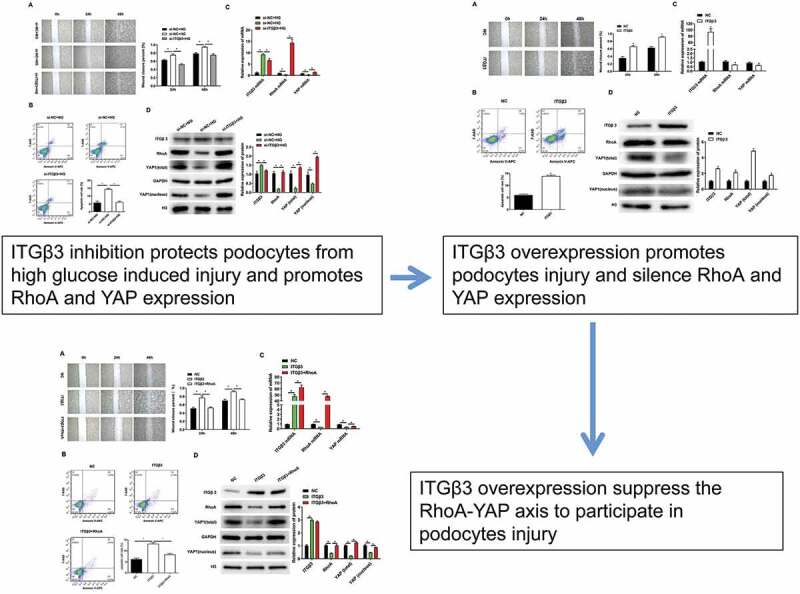

## 1. Introduction

Diabetic nephropathy (DN) has become a major health issue worldwide. Increasing DN accounts for the large proportion of advanced primary renal diseases that has emerged in recent years greatly affects the survival rate and life quality of patients, and causes a significant economic burden to both individuals and the country [1,2]. Hyperglycemia-induced podocytes cellular injury and dysfunction is a main cause of DN [3,4]. Podocytes are a vital component of the glomerular filtration membrane barrier [5–7]. The loss and depletion of podocyte occurs in the early stages of DN, which is often accompanied by a degree of proteinuria [8]. Hence, it is necessary to identify new therapeutic strategies to prevent damage, decrease proteinuria, and improve DN in podocytes.

Integrin β3 (ITGβ3), a member of the cell membrane surface receptors family, regulates the adhesion of cells and transmits molecular signals related to the cellular environment. Studies reported that ITGβ3 plays an important role in gene transcription, cell migration, proliferation, and apoptosis [9,10]. The increasing expression of ITGβ3 is associated with podocyte fusion, skeleton protein damage, podocyte shedding, and proteinuria production, which contributes to the development of DN [11–13]. Inhibition of ITGβ3 by antibody or inhibitor reversed the effect of ITGβ3 overexpression on podocyte injury [13,14]. Our previous study also found that the expression of ITGβ3 was increases and the ability of podocyte would closure was also increased under high glucose (HG) conditions, which was alleviated by spironolactone_ – have been reported reduce proteinuria and delay DN progress [8].

Yes-associated protein (YAP) is an important podocyte transcription cofactor of the downstream Hippo signaling pathway, which regulates the related genes that promote cell proliferation and inhibit apoptosis [15,16]. Studies [9,15,16] found that there is a certain level of YAP in the nucleus of podocytes, which can be used as a receptor for pressure or cytoskeletal signals in the cell microenvironment that are related to podocyte apoptosis. Studies also reported that YAP is an anti-apoptotic molecule in podocytes, and podocytes-specific deletion of YAP results in proteinuria kidney diseases [7,17].

Ras homolog gene family, member A (RhoA), is an important protein involved in the development of various kidney diseases and it is also an important upstream activator of YAP. RhoA regulates many functions of podocytes, including cell morphology and skeleton aggregation, cell proliferation, and apoptosis [18–23]. Kistler AD., Buvall L., and our previous study showed that RhoA is necessary for the normal functioning of glomerular podocytes, silencing or intervening RhoA in podocytes results in loss of tension fibers and production of proteinuria [22,24,25]. The potential mechanism was reducing the expression and nucleation of YAP, thereby causing podocyte cytoskeletal damage and promoting podocyte apoptosis [25].

In addition, steady unidirectional shear stress exert anti-inflammatory and atheroprotective effects by regulating ITGβ3-RhoA-YAP pathway [23]. However, whether ITGβ3 regulates podocyte injury progress by regulating RhoA-YAP axis remains unknown. In this study, we investigate the effect of overexpression and interference of ITGB3 expression on the ability of podocytes in terms of wound closure and apoptosis in the process of podocyte injury, as well as the expression of RhoA and YAP. This will provide a new clue to prevent damage, decrease proteinuria, and improve DN in podocytes.

## 2. Materials and methods

### 2.1 Cell culture and HG exposure

The mouse podocyte cell line MPC5 was purchased from the American Type Culture Collection (Manassas, VA, USA). In addition, MPC5 was cultured according to the manufacturer’s instructions. Briefly, cells were cultured in a Roswell Park Memorial Institute (RPMI)-1640 medium (Gibco, Grand Island, NY, USA) supplemented with 100 U/ml IFN-γ (Cyagen, Guangzhou, China) and incubated with 5% CO_2_ at 33°C for proliferation. Cells were cultured in Dulbecco’s modified Eagle medium (DMEM, Gibco) without IFN-γ for 14 days with 5% CO_2_ at 37°C, which induces differentiation and maturation of podocytes. After differentiation, MPC5 cells were serum-starved for 24 h, and then treated with a normal glucose concentration (NG, 5.3 mM) and HG concentration (HG, 20 mM) for 48 h for further analysis.

### 2.2 Cell transfection

Transfection of MPC5 cells was conducted using Lipofectamine 3000 Reagent (Invitrogen, Carlsbad, CA, USA) according to the manufacturer’s manuals. In brief, when cells reached an 80–90% confluence, transfection was performed. siRNAs or plasmids and Lipofectamine 3000 Reagent were added to the Opti-MEM medium to prepare the siRNA- or plasmid DNA-lipid complexes at room temperature for 15 min. Then the siRNA- or DNA-lipid complexes was added into MPC5 cells. The transfection efficacy was examined by reverse transcription-quantitative polymerase chain reaction (RT-qPCR) and western blot analyses separately after 48 h transfection. siRNAs were synthesized by GenePharma (Shanghai, China), and the sequences were as follows: ITGβ3 siRNA1 (si-ITGβ3-1), sense: 5′-CCUGUUACAAUAUGAAGAAUG-3′, antisense: 5′-UUCUUCAUAUUGUAACAGGGG-3′; ITGβ3 siRNA2 (si-ITGβ3-2), sense: 5′-CAAUGAAGAAGUGAAGAAACA-3′, antisense: 5′-UUUCUUCACUUCUUCAUUGAA-3′; siRNA negative control (si-NC): sense: 5′-UCUCCGAACGUGUCACGUTT-3′, antisense: 5′-ACGUGACACGUUCGGAGAATT-3′. The ITGβ3 was cloned into p HBAd-MCMV-GFP, which was provided by Hanbio (Guangzhou, China), RhoA was clone into pcDNA3.1, which was provided by General Biol (Anhui, China).

### 2.3 RNA extraction and RT-qPCR analysis

The total RNA was extracted from MPC5 cells with TRIzol (Invitrogen) and was converted into complementary DNA (cDNA) using the PrimeScript™ RT Regent kit (Takara, Tokyo, Japan). RT-qPCR analysis was carried out with a Bio-Rad CFX96 Touch q-PCR system (Bio-Rad Laboratories, Inc., Hercules, CA, USA) using Power SYBR Green PCR Master Mix (Roche, Basel, Switzerland) with cDNA and primer pairs (Invitrogen). The mRNA expression was calculated by the 2^−ΔΔCt^ method, and glyceraldehyde-3-phosphate dehydrogenase (GAPDH) was used as the internal control. Primer sequences were as follows: ITGβ3 forward, 5′-TTGGCTACAAACACGTGCTG-3′ and reverse, 5′-CTGCATGATGGCATCAAAGC-3′; RhoA forward, 5′-CAGAAAAGTGGACCCCAGAA-3′ and reverse, 5′-GCAGCTCTCGTAGCCATTTC-3′; YAP forward, 5′-GCAACTCCAACCAGCAGCAACA-3′ and reverse, 5′-CGCAGCCTCTCCTTCTCCATCTG-3′; GAPDH forward, 5′-GAGTCAACGGATTTGGTCGT-3′ and reverse, 5′-GACAAGCTTCCCGTTCTCAG-3′.

### 2.4 Western blot analysis

The nucleus protein was extracted followed the kit instructions (Beyotime Biotechnology, Shanghai, China). The total protein was extracted using a radioimmunoprecipitation assay (RIPA, Beyotime Biotechnology) buffer that contained a protease inhibitor (Beyotime Biotechnology). The supernatants were collected through centrifugation, and an Enhanced BCA Protein Assay Kit (Beyotime Biotechnology) was used to calculate the protein. Subsequently, 30 μg protein in each sample were separated with 10% sodium dodecyl sulfate-polyacrylamide gel electrophoresis (SDS-PAGE) and transferred into polyvinylidene fluoride (PVDF). Then, the PVDF was incubated in 5% fat-free milk for 1 h at room temperature (Thermo, Waltham, MA, USA) and in primary antibody at 4°C overnight. The antibodies were as follows: ITGβ3 (#ab182773, abcam, Cambridge, MA, USA), GAPDH (#60004-1-lg, Proteintech, Wuhan, China), YAP (#4912, Cell Signaling Technology, Danvers, MA, USA), RhoA (#ab187027, abcam), and Histone H3 (#4499s, Cell Signaling Technology). After that, the membrane was incubated with secondary antibody (Proteintech) for 1 h at room temperature. Finally, ECL Plus Substrate (Thermo) and Tanon-5200CE (Biotanon, Shanghai, China) were used to observe the target protein bands.

### 2.5 Flow cytometry analysis

MPC5 cells were collected in phosphate-buffered saline (PBS), washed with a fluorescence-activated cell sorter (FACS) buffer and stained with annexin V-APC and 7-AAD (BD Biosciences, San Jose, CA, USA) in the dark for 30 min at 4°C. Subsequently, cells were washed once with FACS buffer and were resuspended. Then, 10^4^ cells were obtained using a FACScan Flow Cytometer (BD Biosciences, San Jose, CA, USA). The analysis was performed using FlowJo Software 7.6 (TreeStar, Ashland, OR, USA). Apoptotic cells were expressed as a percentage of the total cells.

### 2.6 Wound healing analysis in vitro assay

After transfection, podocytes MPC5 (1 × 10^5^/ml) were grown on six-well dishes overnight and then treated with and without HG. Each well was scratched with a sterile 200-µl pipette tip, washed with PBS, and placed into a fresh medium. Images were acquired by a phase-contrast Leica SP5-FCS microscope (Leica Microsystems, Wetzlar, Germany) at 0, 24, and 48 h after scratching. The wound closure was measured using Image J (National Institutes of Health, Bethesda, MD, USA).

### 2.7 Statistical analysis

Results are expressed as the mean ± standard deviation (SD). Statistical analysis was performed using SPSS 19.0 (SPSS Inc., Chicago, IL, USA) with student’s t-test or analysis of variance followed by Bonferroni post-hoc test. P < 0.05 was determined as the significant level.

## 3. Results

ITGβ3 expression, cell apoptosis, and the ability of wound closure was increased after HG treatment in podocytes. The silence of RhoA also promoted podocyte apoptosis by reducing the expression and nucleation of YAP. Steady unidirectional shear stress exerts an anti-inflammatory and atheroprotective effect through the regulation of the ITG3-RhoA-YAP pathway. However, whether ITGβ3 regulates podocytes injury progression by regulating the RhoA-YAP axis remains unknown. In this study, we investigated the effect of overexpression and interference of ITGB3 on the podocytes’ ability of wound closure and apoptosis as well as the expression of RhoA and YAP. These findings are expected to provide a new insight toward the prevention of podocyte damage, decreased proteinuria, and improved DN.

### 3.1 Test the efficiency of overexpressed ITGβ3 vector and ITGβ3 siRNAs

**Figure 1.**
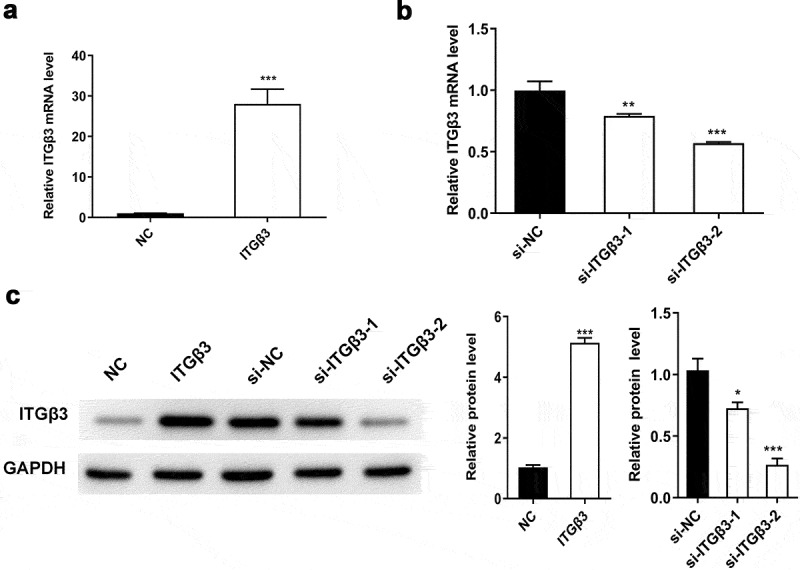
The efficiency of overexpressed ITGβ3 vector and ITGβ3 siRNAs was evaluated in mouse podocyte cell line MPC5. (a) ITGβ3 expression in NC and ITGβ3 group was tested by RT-qPCR, (b) ITGβ3 expression in si-NC, si-ITGβ3-1 and si-ITGβ3-2 group was detected by RT-qPCR. (c) ITGβ3 protein expression was detected by Western blot analysis. Data are shown as mean ± standard deviation, *N* = 3. *, *p* < 0.05; **, *p* < 0.01; ***, *p* < 0.001

Abbreviations: NC: negative control of ITGβ3 overexpression vector; ITGβ3: the group of transfection with integrin β3 overexpression vector; si-NC: small interfering negative control; si-ITGβ3-1: small interfering integrin β3 RNA-1; si-ITGβ3-2: small interfering integrin β3 RNA-2.

At the beginning, we tested the efficiency of overexpressed ITGβ3 vector and ITGβ3 siRNAs on the expression of ITGβ3 through RT-qPCR and western blot analysis after 48 h transfection in mouse podocyte cell line MPC5. Compared with negative control of overexpressed ITGβ3 vector (NC) group, the expression of ITGβ3 in the ITGβ3 group was significantly upregulated (Figure 1a). Meanwhile, si-ITGβ3-1 and si-ITGβ3-2 significantly decreased the expression of ITGβ3, in which si-ITGβ3-2 appeared to be more efficient in inhibiting ITGβ3 expression than si-ITGβ3-1 (Figure 1b). Western blot analysis also confirmed the RT-qPCR results (Figure 1c). Therefore, si-ITGβ3-2 was used for the following experiments to inhibit ITGβ3 expression. The above results verified the overexpressed ITGβ3 vector and ITGβ3 siRNAs was constructed successfully.

### 3.2 Effects of ITGβ3 inhibition on podocytes under HG treatment

**Figure 2.**
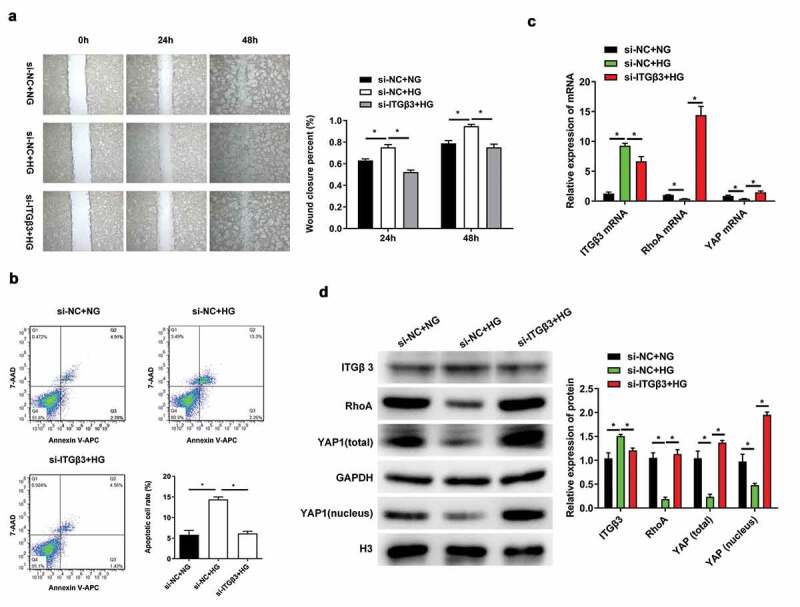
Effects of ITGβ3 inhibition on mouse podocyte cell line MPC5 with NG or HG treatment. (a) Wound healing analysis in vitro assay was used to evaluate the ability of wound closure in MPC5 cells with NG or HG treatment after transfection with si-NC or si-ITGβ3. (b) Apoptosis analysis was detected by Flow cytometry in MPC5 cells with NG or HG treatment after transfection with si-NC or si-ITGβ3. (c) The ITGβ3, RhoA, and YAP mRNA expressions was analyzed by RT-qPCR in MPC5 cells with NG or HG treatment after transfection with si-NC or si-ITGβ3. (d) The ITGβ3, RhoA, total YAP, nuclear YAP protein expression was analyzed by western blot analysis in MPC5 cells with NG or HG treatment after transfection with si-NC or si-ITGβ3. Data are shown as mean ± standard deviation, *N* = 3. *, *p* < 0.05

Abbreviations: ITGβ3: Integrin β3; RT-qPCR: reverse transcription-quantitative polymerase chain reaction; RhoA: Ras homolog gene family, member A; YAP: Yes-associated protein; si-NC: small interfering negative control; si-ITGβ3: small interfering integrin 3 RNA; NG: normal glucose (5.3 mM); HG: high glucose (20 mM); si-NC+NG: MPC5 cells with normal glucose condition treatment after transfection with si-NC; si-NC+HG: MPC5 cells with high glucose condition treatment after transfection with si-NC; si-ITGβ3+ HG: MPC5 cells with high glucose condition treatment after transfection with si-ITGβ3.

In our previous study, ITGβ3 expression was upregulated and the ability of wound closure in vitro and cell apoptosis was promoted under HG situation [8]. Therefore, we had silenced ITGβ3 expression in podocytes, and then investigated the ability of wound closure in vitro, changes in apoptosis, and RhoA and YAP expression after exposure to HG conditions. The in vitro wound healing assay showed that the wound closure ability of podocytes was accelerated after exposure to HG for 24 and 48 h, which was consistent with our previous study [8]. However, ITGβ3 knockdown decreased wound closure ability of podocytes under HG conditions (Figure 2a). During flow cytometry analysis, we observed that the apoptosis of podocytes increased after exposure to HG, whereas the apoptosis was decreased after ITGβ3 expression was silenced under the HG treatment (Figure 2b). These results indicated that HG exposure promotes apotosis and the in vitro wound closure ability in podocytes, whereas the inhibition of ITGβ3 decreases HG-induced the in vitro wound closure ability and apoptosis.

We subsequently investigated whether changes in ITGβ3 expression affect the expression of RhoA and YAP. As RT-qPCR results presented in Figure 2c, ITGβ3 expression was significantly increased in HG-treated podocyes along with the downregulation of RhoA and YAP compared with the NG-treated podocyes after transfection with si-NC. However, RhoA and YAP were upregulated in podocytes under HG conditions when ITGβ3 was inhibited. The same expression trend were also confirmed by western blot analysis (Figure 2d). ITGβ3 expression were upregulated, whereas RhoA, total YAP, and intranuclear YAP expression were downregulated when podocytes transfected with si-NC and were exposed to the HG environment. ITGβ3 knockdown upregulated the expression of RhoA, total YAP, and intranuclear YAP under HG conditions (Figure 2d). The experimental results show that HG promoted the upregulation of ITGβ3 expression in podocytes, along with decreased RhoA, total YAP, and intranuclear YAP expression. However, interfered with the expression of ITGβ3 reversed RhoA, total YAP, and intranuclear YAP expression under HG treatment.

### 3.3 Effects of ITGβ3 overexpression on podocytes

**Figure 3.**
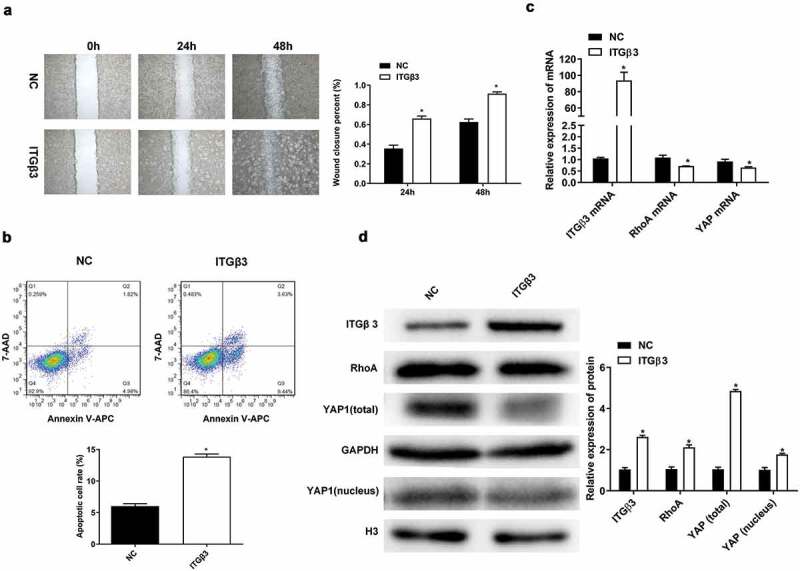
Effects of ITGβ3 overexpression on mouse podocyte cell line MPC5. (a) Wound healing analysis in vitro assay was used to detect the ability of wound closure in MPC5 cells after transfection with NC or ITGβ3. (b) Flow cytometry was used to test apoptosis in MPC5 cells after transfection with NC or ITGβ3. (c) RT-qPCR analysis was used to evaluate ITGβ3, RhoA, and YAP mRNA expressions in MPC5 cells after transfection with NC or ITGβ3. (d) Western blot analysis was used to evaluated ITGβ3, RhoA, total YAP, and nuclear YAP protein expression in MPC5 cells after transfection with NC or ITGβ3. Data are shown as mean ± standard deviation, *N* = 3. *, *p* < 0.05

Abbreviations: NC: negative control of ITGβ3 overexpression vector; ITGβ3: integrin β3; RT-qPCR: reverse transcription-quantitative polymerase chain reaction; RhoA: Ras homolog gene family, member A; YAP: Yes-associated protein.

We also conducted a series of experiments using ITGβ3 overexpression to investigate the wound closure ability of podocytes in vitro, changes in apoptosis, and RhoA and YAP expressions. The wound closure rate of podocytes cells MPC5 was accelerated at 24 and 48 h in the ITGβ3 overexpression group compared to the NC group (Figure 3a), which suggests that ITGβ3 overexpression promotes the ability of podocyte wound closure in vitro. Podocyte apoptosis also increased because of the overexpression of ITGβ3 when compared to NC group (Figure 3b). The result indicated that the overexpression of ITGβ3 contributes to the ability of wound closure and apoptosis in podocytes. In addition, compared with the NC group, the expressions of RhoA and YAP were downregulated when ITGβ3 was overexpression (Figure 3c). These results are also confirmed by western blot analysis shown in Figure 3d, the overexpression of ITGβ3 is associated with the downregulation of RhoA, total YAP, and intranuclear YAP expression. The above results indicate that overexpression of ITGβ3 also decreases the expressions of RhoA, total YAP, and intranuclear YAP in podocytes.

### 3.4 ITGβ3 regulates the RhoA-YAP pathway to participate in podocyte injury progression

**Figure 4.**
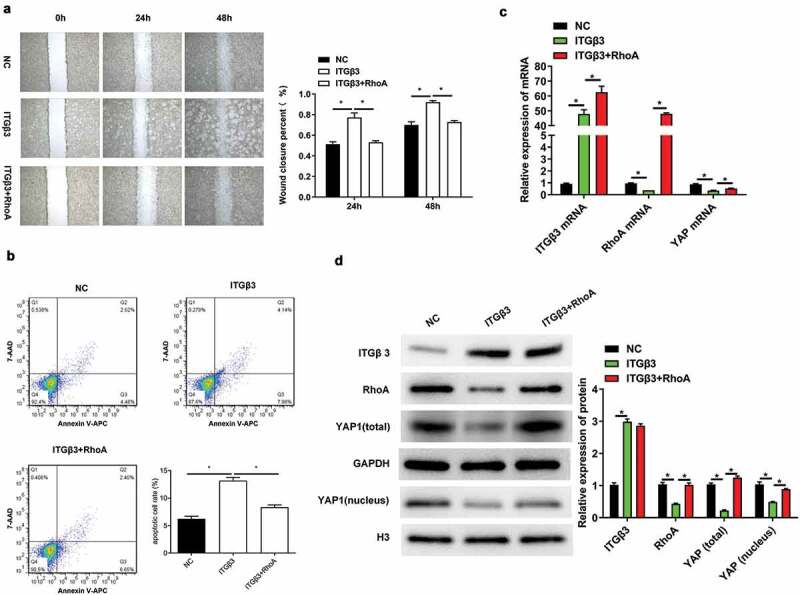
ITGβ3 overexpression inhibits the RhoA-YAP pathway in order to promote podocyte injury progression. (a). Wound healing analysis in vitro assay was used to determine the ability of wound closure in MPC5 cells after transfection with NC, ITGβ3, or ITGβ3+ RhoA. (b). Flow cytometry was used to test apoptosis in MPC5 cells after transfection with NC, ITGβ3 or ITGβ3+ RhoA. (c). RT-qPCR was used to evaluate ITGβ3, RhoA, and YAP mRNA expressions in MPC5 cells after transfection with NC, ITGβ3 or ITGβ3+ RhoA. (d). Western blotting was used to determine ITGβ3, RhoA, total YAP, and nuclear YAP in MPC5 cells after transfection with NC, ITGβ3 or ITGβ3+ RhoA. Data are shown as mean ± standard deviation, *N* = 3. *, *p* < 0.05; NC: negative control of ITGβ3 overexpression vector; ITGβ3: integrin β3; ITGβ3+ RhoA: the group of cotransfection with integrin β3 overexpression vector and Ras homolog gene family, member A overexpression vector

Abbreviations: RT-qPCR: reverse transcription-quantitative polymerase chain reaction; RhoA: Ras homolog gene family, member A; YAP: Yes-associated protein.

To further confirm whether ITGβ3 regulates the RhoA-YAP pathway in podocytes damage, the RhoA overexpression vector was synthesized, and cotransfection with ITGβ3 overexpression vector into MPC5 cells was performed. The efficiency of overexpressed RhoA vector was confirmed by RT-qPCR and western blotting. The results demonstrated that RhoA expression was significantly increased when ITGβ3 was overexpressed in MPC5 cells compared to that in the case of only transfection with the ITGβ3 overexpression vector (Figure 4c and d). Based on the results of Figure 3c and d; Figure 4c and d, the overexpression of ITGβ3 in MPC5 cells significantly decreases the expression of RhoA, and the expression of RhoA is increased after transfection with RhoA overexpression vector in ITGβ3 overexpression MPC5 cells, these results revealed that the RhoA overexpression vector was constructed successfully. Then, the ability of wound closure was measured in MPC5 cells through an in vitro wound healing analysis. As shown in Figure 4a, the RhoA overexpression attenuated the effect of ITGβ3 overexpression on the ability of wound closure. In addition, RhoA overexpression partially reversed apoptosis in ITGβ3 overexpression-induced podocytes apoptosis through flow cytometry analysis (Figure 4b). Furthermore, the RhoA overexpression upregulated YAP expressions when compared with those of the ITGβ3 overexpression group (Figure 4c). This change was also observed in western blotting analysis. RhoA overexpression reversed the inhibition effect of ITGβ3 overexpression on the expression of total YAP and intranuclear YAP in MPC5 cells (Figure 4d). The above results indicate that ITGβ3 overexpression promotes podocytes injury by inhibiting the RhoA-YAP pathway.

**Figure 5.**
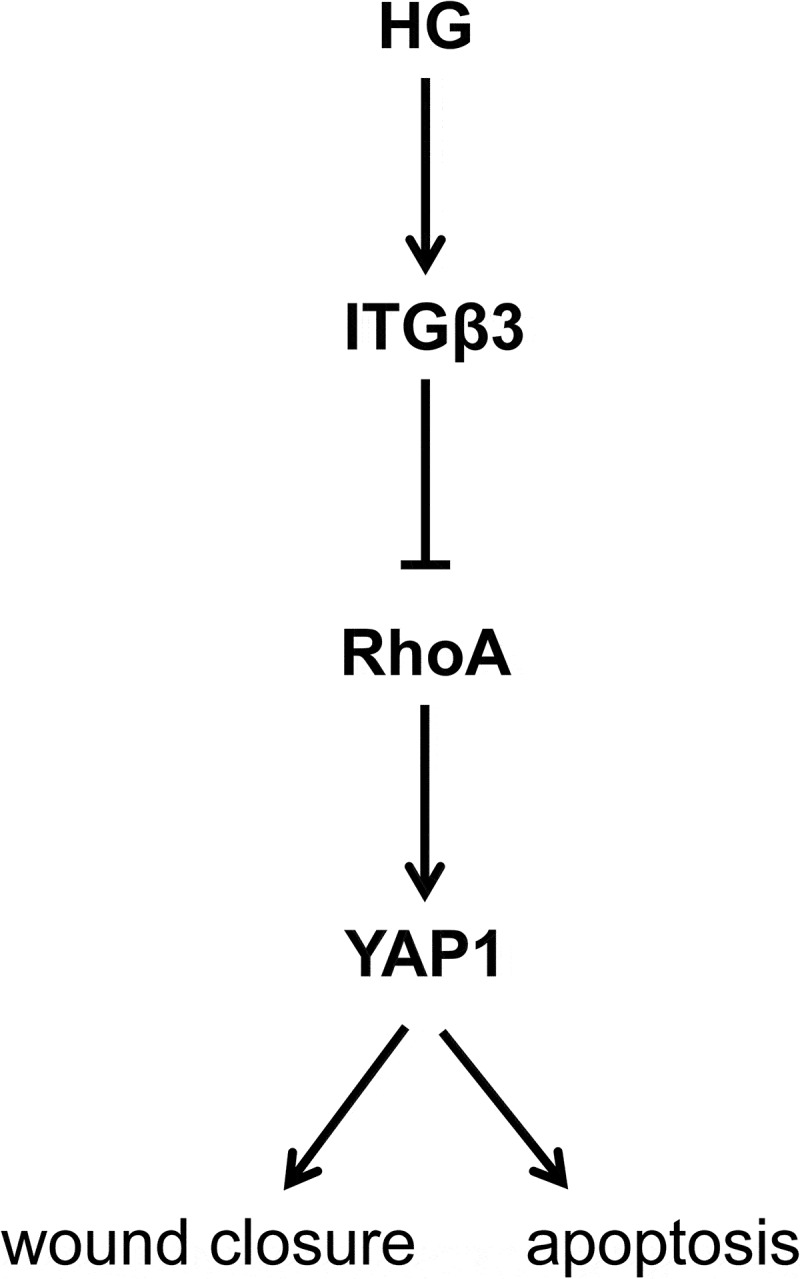
A schematic to unveil our work in this study. HG-induced ITGβ3 overexpression inhibited the expression of RhoA-YAP1 axis, resulting in the promotion of the ability of wound closure in vitro and podocyte apoptosis

Abbreviations: HG: high glucose (20 mM); ITGβ3: integrin 3; RhoA: Ras homolog gene family, member A; YAP: Yes-associated protein.

In addition, a schematic was drawn to unveil our findings in the present study. As shown in Figure 5, HG induced the expression of ITGβ3 overexpression, which consequently inhibited the expression of RhoA-YAP1 axis, which resulted in the promotion of the ability of wound closure and apoptosis.

## 4. Discussion

DN has become a serious complication in diabetics with the increasing trend of diabetes diagnoses in recent years [1,26]. Podocytes are a vital component of the glomerular filtration membrane barrier, and previous studies indicate that the damage of podocytes is one of the important pathological mechanisms of DN [5,6,27]. Our previous study revealed that the ITGβ3 expression was upregulated, and the ability of wound closure and cell apoptosis was promoted in podocytes in HG conditions [8]. A previous study also demonstrated the silence of RhoA-promoted podocyte apoptosis via reduction of the expression and nucleation of YAP [25]. Furthermore, steady unidirectional shear stress exerted anti-inflammatory and atheroprotective effects by regulating the ITGβ3-RhoA-YAP pathway [23]. Whether ITGβ3 regulates podocytes injury progression by regulating the RhoA-YAP axis is the main purpose of the present study.

In this study, we found that the ability of wound closure in vitro and cell apoptosis was promoted and that the expression of RhoA, total YAP, and intranuclear YAP were inhibited in podocytes under HG exposure; the silence of the expression of ITGβ3 in MPC5 podocytes attenuated the effect of HG on the ability of wound closure and cell apoptosis, increased the expression of RhoA, total YAP, and intranuclear YAP in HG-treated MPC5 podocytes. In addition, transfection with ITGβ3 overexpression vector promoted the ability of wound closure in vitro and cell apoptosis along with the inhibition of the expression of RhoA, total YAP, and intranuclear YAP. However, RhoA overexpression reversed the effects of ITGβ3 overexpression on the ability of wound closure and cell apoptosis as well as rescued the expression of total YAP and intranuclear YAP. These results together indicated that the ITGβ3 overexpression regulated podocyte injury by inhibiting the RhoA–YAP pathway.

Normal podocytes express low levels of ITGβ3; however, increasing ITGβ3 was expressed in podocytes under certain pathological circumstances, including diabetes. Similarly, the expression level of ITGβ3 increased, accompanied by podocyte fusion, skeleton protein damage, podocyte shedding, and proteinuria. Increased ITGβ3 expression was observed in focal segmental glomerulosclerosis (FSGS) and diabetic patients, as well as in LPS-induced nephropathy rat models [11–13]. An in vitro study found that increased ITGβ3 is associated with enhanced podocytes motility [8]. Increased urokinase receptor (uPAR) was observed in the HG-exposed podocytes [28]. Previous studies found that ITGβ3 interacts with uPAR in podocytes and produces a complex, which results in a structural modification of ITGβ3 and enhances its activation [29,30]. Inhabiting the expression of ITGβ3 was confirmed to be linked to an improvement in podocytes foot and anti-proteinuric effects in an in vivo study using a mice model [31]. These results suggest that ITGβ3 plays an important role in the development of kidney disease. Our study observed that ITGβ3 inhibition contributes to depressing the ability of wound closure of podocytes in vitro during HG-induced injury, and a high expression of ITGβ3 promotes the ability of wound closure of podocytes in vitro; these findings are consistent with previous findings. As HG-induced podocytes injury is a main pathogenesis during the progression of DN, our findings added extra evidence that ITGβ3 is associated with DN and is likely to be a promising target for DN treatment.

RhoA is a Rho GTPases and has been considered to be a main regulator of the cytoskeletal architecture function [32,33]. The baseline activity of RhoA plays an important role in the normal function of glomerular podocytes. A previous study indicated that RhoA stabilizes the foot process structure of podocytes, thereby preventing proteinuria and related renal disease; however, inhibition of RhoA increases the motility of podocytes and results in proteinuria [22,34]. Consistent with previous findings, we found that RhoA was decreased in HG-treated podocytes, the ability of wound closure in vitro was increased at the same time. However, silence of ITGβ3 expression promoted the expression of RhoA, and attenuate the effect of HG treatment on the ability of wound closure in vitro. This result suggests that ITGβ3 regulates podocyte injury by inhibiting the expression of RhoA.

YAP is an important podocyte transcription cofactor of the downstream Hippo signaling pathway, which regulates the related genes that promote cell proliferation and inhibit apoptosis [15,16]. Cdc42/Nwasp/F-actin is an important signaling pathway that regulates cytoskeletal polymerization, and YAP mediates podocyte damage and apoptosis caused by the loss of Cdc42/Nwasp/stress fiber [35–38]. Silencing YAP causes significant damage and the apoptosis of podocytes, which suggests that YAP overexpression protects podocytes from damage [35,39]. In addition, RhoA deficiency and the loss reduces the expression and nucleation of YAP, thereby causing podocyte cytoskeletal damage and promoting podocyte apoptosis [7,17,25]. Furthermore, steady unidirectional shear stress exerted anti-inflammatory and atheroprotective effects be regulating the ITGβ3–RhoA–YAP pathway [23]. In this study, we observed that ITGβ3 overexpression is associated with the downregulation of RhoA, YAP, total YAP, and nuclear YAP, which increases the apoptosis and the ability of wound closure of podocytes. However, these can be reversed by the overexpression of RhoA, which mitigates the harm to podocytes. To the best of our knowledge, our study was the first time to suggest that ITGβ3 overexpression increased the damage to the podocytes by regulating the RhoA–YAP pathway, which provides novel clue for DN treatment.

However, more studies are needed to state the way of how ITGβ3 regulates RhoA-YAP pathway, and ITGβ3-RhoA-YAP axis also need verified in vivo animal model.

## 5. Conclusion

Taken together, we found that ITGβ3 inhibition contributes to the protection of podocytes from damage. In addition, ITGβ3 overexpression inhibits RhoA-YAP activation, thereby promoting the HG-induced apoptosis and the ability of wound closure of podocytes in vitro. In other words, ITGβ3 overexpression promotes podocytes injury by inhibiting the RhoA–YAP pathway. These findings add new evidence for the important role of ITGβ3 in regulating podocyte injury in DN patients and highlights new insights into the molecular pathogenesis of DN.

## Highlights

1. High glucose treatment promotes ITGβ3 expression.

2. High glucose treatment promotes podocyte damage.

3. ITGβ3 inhibition protects podocytes from high glucose-induced injury.

4. ITGβ3 overexpression suppresses RhoA–YAP axis contributes to podocyte injury.
